# Relationship between muscle strength and fall episodes among the elderly: the Yilan study, Taiwan

**DOI:** 10.1186/s12877-018-0779-2

**Published:** 2018-04-13

**Authors:** Nan-Ping Yang, Nai-Wei Hsu, Ching-Heng Lin, Hsi-Chung Chen, Hsuan-Ming Tsao, Su-Shun Lo, Pesus Chou

**Affiliations:** 1 Community Medicine Research Center & Institute of Public Health, National Yang-Ming University, No.155, Sec.2, Linong Street, Taipei, 112 Taiwan, Republic of China; 2 Department of Orthopedics & Surgery, Keelung Hospital, Ministry of Health & Welfare, Keelung, Taiwan; 3 Department of Intern Medicines, National Yang-Ming University Hospital, Yilan, Taiwan; 4 Department of Medical Research, Taichung Veterans General Hospital, Taichung, Taiwan; 5 Department of Psychiatry & Center of Sleep Disorders, National Taiwan University Hospital, Taipei, Taiwan; 6 Department of Surgery, National Yang-Ming University Hospital, Yilan, Taiwan

**Keywords:** Hand grip strength, Falling, Elderly population

## Abstract

**Background:**

Fall episodes are not unusual among community residents, especially the elderly, and lower muscle strength is an important issue to address in order to prevent falls.

**Methods:**

A community health survey was conducted in a suburban area of Taiwan, and 1067 older adults were selected for enrollment in the present study. All the enrolled subjects had been visited at their homes; the subjects’ strength of both hands and muscle mass of both legs were measured and well-established questionnaires were finished by certificated paramedic staffs.

**Results:**

The incidence of fall episodes in the previous 1 year in the Yilan elderly population was 15.1%, and the female predominance was significant. A significantly higher prevalence of cataracts was found in group who experienced a fall in the past year (64% vs. 54.9% in the non-fall group). Mild or more severe dementia was much more prevalent in the group who experienced a recent fall (33.8% vs. 25.7% in the non-fall group). The strength of both hands tested as the physical function was 17.6 ± 8.0 kg in the recent fall group, significantly weaker than that in the non-fall group (20.7 ± 8.7 kg). Multivariate regression analysis revealed a greater weekly exercise duration and greater strength of both hands reduced the occurrence of falls among the whole and the female population. The standardized effect sizes of hand grip strength between both groups, not trivial, were 0.29 and 0.37 for the total population and the female subpopulation respectively.

**Conclusions:**

Less weekly exercise duration and weaker muscle strength were f ound to be independent risk factors of fall episode(s) in an elderly Taiwanese population, especially in the female sub-population. Muscle strength, measured by average of both hands grip strength, was the most significantly factor of one-year fall episode(s) accessed retrospectively.

## Background

Fall episodes were not unusual in community residents, especially the elderly. Moreover, approximately 30% of people over 65 years of age living in the community fall each year, according to a review based on the Cochrane database [1]. Injuries sustained in a fall were more likely to be serious than those due to other reasons in community-dwelling older adults that were characterized as hip fracture, head injury and/or other fracture [2]. From viewpoints of prevention medicine, all trials to reduce rate of falling or consequence of falling would be worthy. For an example, environmental improvements such as impact-absorbing flooring could significantly reduce the relative risk (RR) of injury in the event of a fall by 59% among the frail elderly in a nursing home [3]. Moreover, many studies had aimed to develop various exercise programs to reduce the risk of falling in older adults [1, 4]. Therefore, exercise was expected to be of benefit to prevent falls and even subsequent injuries.

.Poor lower extremity physical function, especially a poor walking ability, has been found to be an important risk factor related to falls in elderly community-dwelling people [5]. Usually, walking ability can be measured quantitatively by some timed walking tests such as Timed-Up & Go [TUG], 10-m walk test [10MWT], 6-min walk test [6]. The predictive value of walking speed as an individual physical frailty indicator on activities of daily living (ADL) disability had been approved in community-dwelling elderly people [7] and, some elderly persons having low physical function and low self-confidence in terms of balance tend to overestimate their balance and ambulation function that might have a high risk of accidents [8]. However, in terms of a large-scale community survey, it would be necessary to find some more feasible measures rather than the above-mentioned functional tests that could be performed quickly and safely by the older population. There were two objectives could be considered.

First, low muscle mass (i.e. sarcopenia) could be defined as age-related lean tissue mass loss that usually resulted in reduced physical function, muscular strength, and mobility [9]. Reported prevalence of sarcopenia ranged widely depending on the reference cut-off thresholds for muscle mass and calf circumference; racial and geographic differences should be also taken into considerations [10]. Recently, sarcopenia could be defined using the Asian Working Group consensus algorithm, combining muscle mass, gait speed, and even grip strength and, adjusted by age and gender [11]. Second, low muscle strength was associated with poor physical function in older people, both male and female [12]. Hand grip strength was shown to be correlated with sarcopenia, physical function and walking ability [13, 14]. Additionally, grip strength was also a simple and inexpensive test, positively correlated with measures of nutritional health [15] and could be considered in clinical settings for identifying future falls and even fractures [15, 16].

It was rarely studied to investigate the influences of exercise, muscle mass, and muscle strength on falling, especially in an Asia elderly population. As a result, this study was a community-based survey to study the relationships between the above mentioned indicators (exercise, muscle mass, muscle strength) and fall episode(s) in an elderly Taiwanese population.

## Methods

### Study design and participants

The study was part of the Yilan study [17, 18], a population-based community health survey conducted by the Community Medicine Research Center at the National Yang-Ming University and the National Yang-Ming University Hospital in Taiwan. The protocol was evaluated and approved by the Institutional Review Board (IRB) of National Yang-Ming University Hospital (IRB Approval No.: 2011A016), which has been certificated by the Ministry of Health and Welfare, Taiwan. All the enrolled participants provided written informed consent.

From August 2013 to February 2016, all residents aged 65 years or more living in Yilan City, located in northeast Taiwan, were randomly selected to participate. All participated subjects had been visited at their homes, and well-established questionnaires [19], approved by the IRB of National Yang-Ming University Hospital, were used to collect their basic data and health-related information including demographic characteristics, body weight and height, education level, living status, lifestyle, smoking and drinking habits, self-reported medical histories (i.e., history of various chronic diseases), exercise condition within past 1 week, sleep status and quality of life within past 1 month, falling episode within past 1 year and current psychological status (i.e., dementia measured by the ascertain dementia 8-item informant questionnaire (AD8) [20]) that were administered by trained project assistants through face to face interviews. Excluding the individuals who failed to provide a past medical history, who could not complete interview, or who were unable to cooperate with the collection of anthropometric data or physical functional test, finally there were 1067 older adults enrolled in the present study.

### Measurements of hand grip strength and muscle mass

The strength of both hands and the muscle mass of both legs were also measured by certificated paramedic staff of the National Yang-Ming University Hospital in Yilan, Taiwan, using a hydraulic hand dynamometer (Jamar, Jackson, MI, USA) and an InnerScan segmental body composition analyzer (InnerScan V,TANITA® BC601, Japan) with an operating frequency of 50 kHz at 100 μA respectively. Every participant completed two trials for each hand and, the best performance of each hand was averaged as the final estimate of hand grip strength [19]. The estimation of segmental muscle mass for mode BC601 has been validated [21]; the estimated lean mass (kg) for each lower extremity was averaged as the final estimate.

### Statistical analysis

Descriptive statistics were presented as numbers of cases, percentages, and means with standard deviation (SD). The independent t-test and the chi-square test were used to analyze significant differences between groups (group I: population without any fall episode; group II: population with fall episode(s)) for continuous and categorical variables. Adjusted odds ratios (AORs) and 95% confidence intervals (CIs) were calculated by the logistic regression method to perform multivariate analyses. Furthermore, the magnitude of effects (i.e. effect size (ES) statistics) of those significantly effective independent variables was also calculated to assess the relationships within data [22]. The formula of effect size is as below [23]:

$$ \mathrm{Standardized}\ \mathrm{ES}=\left(\left[\mathrm{mean}\ \mathrm{of}\ \mathrm{group}\ 1\right]\hbox{--} \left[\mathrm{mean}\ \mathrm{of}\ \mathrm{group}\ 2\right]\right)/{\mathrm{SD}}_{\left(\mathrm{pooled}\right)} $$

In addition, the Cohen’s *ƒ*^2^ effect size measure for multiple regression is defined as:

$$ {f}^2={R}^2/1-{R}^2, $$

where *R*^2^ is the squared multiple correlation [24].

All analyses of data were conducted using the Statistical Package for Social Sciences for Windows (SPSS Ver.22.0). All reported *p* values are two-tailed, and were considered significant if *p* ≤ 0.05.

## Results

The basic characteristics of all the enrolled subjects are presented in Table 1. The study included 1067 people aged 65 years or more (mean age: 76.4 ± 6.0 years), 439 males (41.1%) and 628 females (58.9%). Only a small proportion of the studied subjects lived alone, 4.4% for part of the time and 6.8% full-time. The prevalence of various chronic physical disorders related to accidental falls, including diabetes mellitus (24.6%), hypertension (58.7%), coronary arterial disease (34.3%), cerebrovascular disease (2.7%) and cataracts (56.2%), were also investigated. Using the clinical dementia rating (CDR) method, evaluated based on AD8, to identify the cognitive impairment status of the elderly subjects, 228 subjects (27.0%) were considered to have mild or more severe dementia. Within 1 year, the incidence of falls, which was accessed retrospectively, in the studied Yilan elderly population, was 15.1%, and their exercise duration was 182 ± 223 min per week. When visited at home, the strength of both hands and the muscle mass of both legs of the enrolled subjects were measured to be 20.2 ± 8.7 and 7.3 ± 1.8 kg, respectively.

**Table 1:** Basic characteristics of the enrolled subjects and their experiences of falls within the past 1 year

	Total (*N* = 1067)	
	No., Mean	%, SD
Gender (male)		
male	439	41.1
female	628	58.9
Age (years)	76.4	6.0
Living Status		
full-time living alone	73	6.8
part-rime living alone	47	4.4
not living alone	947	88.8
Chronic Physical Disorders		
DM (yes)	263	24.6
HT (yes)	626	58.7
CAD (yes)	366	34.3
CVA (yes)	29	2.7
Cataract (yes)	600	56.2
Cognitive Impairment (by CDR score)		
mild or more severe dementia (≧2)	228	27.0
Fall episode within the past year (yes)	161	15.1
Strength (kg), average of both hands	20.2	8.7
Muscle mass (kg), average of both legs	7.3	1.8
Exercise (mins), accumulated in 1 week	182	223

*DM* diabetes mellitus, *HT* hypertension, *CAD* coronary arterial disease, *CVA* cerebrovascular disease

The study population was divided into two subgroups, a fall group (those who had experienced a fall within the past year) and a non-fall group (those who had not experienced a fall in the past year), and the characteristics of the two different populations were compared (as shown in Table 2). A significantly greater proportion of female subjects experienced a fall episode within the past year, accounting for 68.9% of the fall subgroup (as compared with 57.1% of the non-fall group; *p* = 0.005). A significantly higher prevalence of cataracts was also found in the fall group (64%, as compared with 54.9% in the non-fall group; *p* = 0.03). Mild or more severe dementia, as indicated by a CDR score ≥ 2, was much more prevalent in the fall group than the non-fall group (33.8% vs. 25.7%; *p* = 0.05). With regards to the physical function tests, only the strength of both hands differed significantly between groups, at 17.6 ± 8.0 kg in the fall group vs. 20.7 ± 8.7 kg in the non-fall group (*p* < 0.0001).

**Table 2:** Bivariate analysis of cases with fall episode(s) within the past 1 year compared to the others

	Population without any fall episode		Population with fall episode(s)		*P* value of χ^2^ test or t test
	No., Mean	%, SD	No., Mean	%, SD	
Gender					
male	389	42.9	50	31.1	0.005
female	517	57.1	111	68.9	
Age (years)	76.3	6.0	76.8	6.0	0.37
Living Status					
full-time living alone	56	6.2	17	10.6	0.08
part-rime living alone	38	4.2	9	5.6	
not living alone	812	89.6	135	83.9	
Chronic Physical Disorders					
DM (yes)	222	24.5	41	25.5	0.79
HT (yes)	530	58.5	96	59.6	0.79
CAD (yes)	311	34.3	55	34.2	0.97
CVA (yes)	25	2.8	4	2.5	0.84
Cataract (yes)	497	54.9	103	64.0	0.03
Cognitive Impairment (by CDR score)					
mild or more severe dementia (≧2)	183	25.7	45	33.8	0.05
Strength (kg), average of both hands	20.7	8.7	17.6	8.0	< 0.0001
Muscle mass (kg), average of both legs	7.3	1.8	7.1	2.1	0.14
Exercise (mins), accumulated in 1 week	187	226	153	201	0.08

*CDR* clinical dementia rating, *kg* kilograms, *mins* minutes

A multivariate logistic regression model was used to analyze the factors associated with a fall episode within the past year among the studied elderly population of Yilan (Table 3). A greater duration of exercise per week was shown to reduce the occurrence of fall(s) significantly among the whole study population (AOR 0.999, 95% CI 0.998–1.000; standardized ES 0.15), and particularly in the female subgroup (AOR 0.999, 95% CI 0.997–1.000; standardized ES 0.23). A greater strength of average of both hands was also shown to reduce the occurrence of fall(s) significantly among the whole study population (AOR 0.95, 95% CI 0.92–0.98; standardized ES 0.37), and particularly in the female subgroup (AOR 0.94, 95% CI 0.90–0.99; standardized ES 0.29). A small effect of 0.2 is noticeably smaller than medium (ES = 0.5) but not so small as to be trivial [23]. That meant that the elder community-dwelling Taiwanese adults without any fall episode within the past 1 year had a higher score of hand grip strength than 58–69% of the others [23]. Furthermore, the Cohen’s *ƒ*^2^ effect size measures for multiple regressions were calculated that effect size for full model in female population was 0.057 and in total population was 0.038. By convention, *ƒ*^2^ effect sizes of 0.02, 0.15, and 0.35 are termed *small*, *medium*, and *large*, respectively [24]. Similarly, the effect size from the regression models is still noticeably smaller than medium but not so small as to be trivial. Otherwise, among the medical histories, only positive CAD status was an associated factor of falling in the female subpopulation (AOR 0.58, 95% CI 0.33–1.00).

**Table 3:** Multivariate analysis of fall episode(s) within the past 1 year

	Male Population		Female Population			Total Population		
	AOR	95% CI	AOR	95% CI	St. ES^a^	AOR	95% CI	St. ES^a^
Gender (male)	–		–			0.80	0.40–1.58	
Age (years)	1.02	0.95–1.08	0.96	0.92–1.01		0.99	0.95–1.02	
Living Status								
full-time living alone	Ref.		Ref.			Ref.		
part-rime living alone	0.36	0.03–4.18	1.99	0.60–6.57		1.19	0.43–3.32	
not living alone	0.40	0.11–1.38	0.75	0.33–1.68		0.68	0.35–1.32	
Chronic Physical Disorders								
DM (yes)	1.78	0.84–3.76	0.73	0.41–1.32		0.97	0.62–1.51	
HT (yes)	0.91	0.43–1.89	0.89	0.54–1.47		0.90	0.60–1.36	
CAD (yes)	1.08	0.52–2.26	0.58	0.33–1.00		0.68	0.44–1.05	
CVA (yes)	2.38	0.55–10.3	0.26	0.03–2.17		0.85	0.28–2.58	
Cataract (yes)	1.08	0.54–2.15	1.55	0.93–2.57		1.35	0.90–2.02	
Cognitive Impairment (by CDR score)								
mild or more severe dementia (≧2)	1.01	0.47–2.17	1.51	0.89–2.55		1.29	0.85–1.95	
Strength (kg), average of both hands	0.96	0.92–1.01	0.94	0.90–0.99	0.29	0.95	0.92–0.98	0.37
Muscle mass (kg), average of both legs	1.12	0.92–1.38	1.01	0.75–1.37		1.12	0.95–1.32	
Exercise (mins), accumulated in 1 week	1.001	0.999–1.002	0.999	0.997–1.000	0.23	0.999	0.998–1.000	0.15

*AOR* adjusted odds ratio, *CI* confidence interval, *St. ES* standardized effect size, *Ref.* reference group

^a^ effect size for full model in female population and total population = 0.057 and 0.038, respectively

Summarily, muscle strength, measured by average of both hands grip strength, was the most significantly independent factor of one-year fall episode(s) accessed retrospectively among the elderly in a Taiwan’s community.

## Discussion

In Taiwan, an urban community surveillance of residents aged 65 years or over in the capital city showed that 16.3% of the elderly persons interviewed had experienced at least one fall in the past 1 year, and gender, visual impairment and orthostatic hypotension were identified as major risk factors of fall injuries in the elderly [25]. A total of 53.4% of the participants reported a fear of falling [26], and fear of falling was identified as a major factor influencing health-related quality of life in the same elderly population [27]. In the present study, performed in a suburban community in Taiwan, the incidence of falling in the past year was 15.1% with its 95% CI (13.0–17.2%) that was equivalent as compared with the abovementioned previous study.

After systematic reviewing of literatures, group and home-based exercise programs, and home safety interventions were well-known to reduce rate of falls and risk of falling [1]. Furthermore, exercise interventions in community-dwelling older people also could probably reduce fear of falling to a limited extent immediately after the intervention, without increasing the risk or frequency of falls [28]. In the present study, the exercise behavior (i.e. duration of exercise per week) was also found to be a potentially protective factor of falling episode(s) for the community-dwelling older adults in Taiwan although it was not statistically significant (*p* = 0.08) in the stage of bivariate analysis. However, it was still an important factor of falling in the elderly after adjusting other variates in the final multivariate logistic model.

In the present study, another meaningful finding was that mild or more severe dementia, as indicated by a CDR score ≥ 2, was much more prevalent in the fall group than the non-fall group (33.8% vs. 25.7%; *p* = 0.05). In another study in Taiwan, 280 Taiwanese people aged 65–91 years who were not taking anti-depressant medication were followed-up for 2 years with monthly telephone calls to determine the incidence of falls: 19.6% of the participants had experienced a fall on one occasion, and 13.5% had fallen two or more times. Depressive symptoms were significantly more prevalent in the recurrent fallers (40.0%) and the once-only fallers (27.5%) than in the non-fallers (16.1%) [29]. Findings of the two studies in similar geographic areas were still similar.

Furthermore, in the present study, a medical history of cataract was still noted to be a risk factor of falling episode(s) for the community-dwelling older adults in Taiwan in the stage of bivariate analysis. Poor vision was known to reduce postural stability and significantly increase the risk of falls and fractures in older people [30]. A systematic review of the literature concluded that expedited cataract surgery was effective in significantly enhancing vision (OR, 7.22; 95% CI, 3.16–16.55; *P* < .0001) but was inconclusive in preventing falls (OR, 0.81; 95% CI, 0.55–1.17) [31]. It would be worthy to further study the relationship between visual problems and falling in the elderly.

Sarcopenia had been shown to be associated with an increase in the risk of falling or physical disability in community-dwelling middle-aged and older adults in several studies [32–37]. However, in the present study, a cross-sectional survey of suburban community-dwelling elderly people in Yilan, Taiwan, showed that there was no difference in the muscle mass of both legs between the elderly subjects who had and had not experienced a fall in the past year. It would be possible due to the healthy effect because the randomly selected subjects who were unable to cooperate with the collection of anthropometric data or physical functional test were excluded from the present study. Further long-term follow-up study of the subjects enrolled or excluded in the present study and comparisons among urban-, suburban- and rural-community living elderly people are necessary to be assessed in depth.

On the other hand, appendicular skeletal muscle mass was found to be significantly correlated with handgrip strength in elderly Chinese subjects [38]; additionally, hand grip strength had been reviewed and concluded to be used for the diagnosis of sarcopenia and frailty [39]. Focused on older people, many recent studies had concluded that strength measures, including peak torque of flexors and extensors of the knee and hip joints, maximum voluntary isometric strength (MVIS) of hip abductors, and hand grip strength, were better than muscle mass measures in terms of predicting health-related outcomes such as balance, functional mobility, daily physical activities and falls [40–43]; both hand grip strength and knee extension strength, highly correlated and internally consistent, were commonly used to measure muscle power [44, 45]. Many measured physical tests, including grip strength (GS), the chair stand test (CST), one-leg standing test (OLS), TUG, 10MWT, 6-m walking speed at a comfortable pace (CWS) and at maximum pace (MWS), and TUG, even self-reported walking ability, had been used to screen fall risk and functional activities in frail older people [6, 46–48]. Summarily, measurements of both hand grip forces by the dynamometry were more convenient and well established as an indicator of muscle status, particularly among older adults. In the present study, the strength of both hands was significantly weaker in the fall group (17.6 ± 8.0 kg) than the non-fall group (20.7 ± 8.7 kg, *p* < 0.0001). At the final multivariate logistic model, hand strength was the most significantly independent factor of one-year fall episode(s). This finding should encourage health promotion activities for the elderly in communities in Taiwan and other countries. Therefore, in terms of a large-scale community survey such as the present study, hand grip strength would be the most feasible measure in the older population rather than other functional test.

## Conclusion

The prevalence of fall episodes in the past 1 year in the Yilan elderly population was 15.1%, and a shorter weekly exercise duration and weaker strength of both hands were independent risk factors related to falls in the total population and in the female sub-population. Especially, muscle strength, measured by average of both hands grip strength, was the most significantly independent factor of one-year fall episode(s) accessed retrospectively among the elderly in a Taiwan’s community.

## Abbreviations

10MWT: 10-m walk test
AD8: Ascertain dementia 8-item informant questionnaire
ADL: Activities of daily living
AOR: Adjusted odds ratios
CDR: Clinical dementia rating
CIs: Confidence intervals
CST: Chair stand test
CWS: Walking speed at a comfortable pace
ES: Effect size
EWGSOP: European working group on sarcopenia in older people
GS: Grip strength
IRB: Institutional review board
MVIS: Maximum voluntary isometric strength
MWS: Walking speed at maximum pace
OLS: One-leg standing test
RR: Relative risk
SD: Standard deviation
TUG: Timed up-and-go test
